# A data security scheme based on EEG characteristics for body area networks

**DOI:** 10.3389/fnins.2023.1174096

**Published:** 2023-05-18

**Authors:** Tong Bai, Yuhao Jiang, Jiazhang Yang, Jiasai Luo, Ya Du

**Affiliations:** ^1^School of Optoelectronic Engineering, Chongqing University of Posts and Telecommunications, Chongqing, China; ^2^The Women and Children Hospital of Yongchuan, Chongqing, China; ^3^Department of Peripheral Vascular (Wound Repair), Chongqing Hospital of Traditional Chinese Medicine, Chongqing, China

**Keywords:** body area network, electroencephalogram, data security, wavelet packet transform, linear feedback shift register

## Abstract

Body area network (BAN) is a body-centered network of wireless wearable devices. As the basic technology of telemedicine service, BAN has aroused an immense interest in academia and the industry and provides a new technical method to solve the problems that exist in the field of medicine. However, guaranteeing full proof security of BAN during practical applications has become a technical issue that hinders the further development of BAN technology. In this article, we propose a data encryption method based on electroencephalogram (EEG) characteristic values and linear feedback shift register (LFSR) to solve the problem of data security in BAN. First, the characteristics of human EEG signals were extracted based on the wavelet packet transform method and as the MD5 input data to ensure its randomness. Then, an LFSR stream key generator was adopted. The 128-bit initial key obtained through the message-digest algorithm 5 (MD5) was used to generate the stream key for BAN data encryption. Finally, the effectiveness of the proposed security scheme was verified by various experimental evaluations. The experimental results showed that the correlation coefficient of data before and after encryption was very low, and it was difficult for the attacker to obtain the statistical features of the plaintext. Therefore, the EEG-based security scheme proposed in this article presents the advantages of high randomness and low computational complexity for BAN systems.

## 1. Introduction

**Figure 1:**
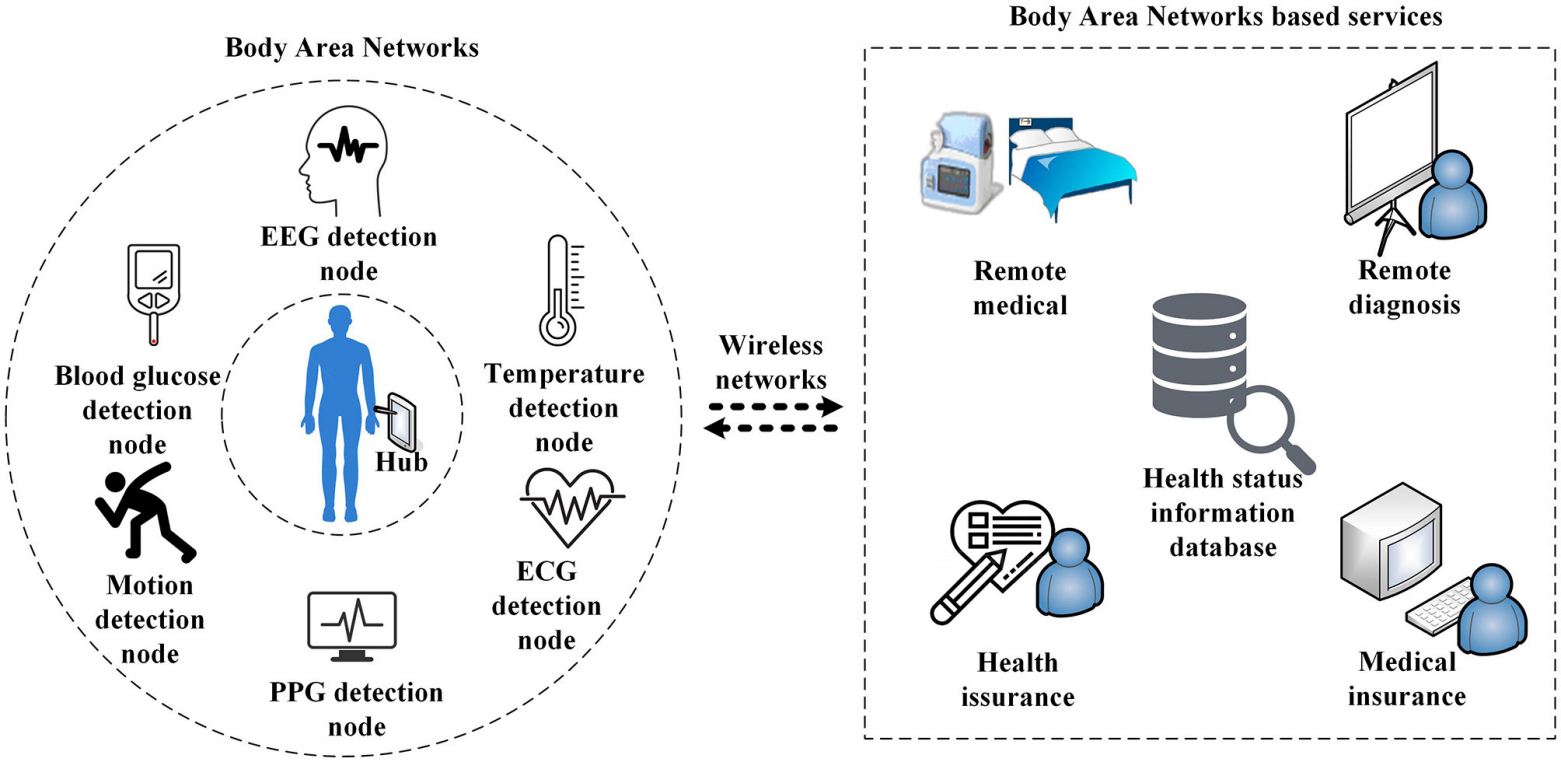
Body area network (BAN) system and its applications.

Body area network (BAN) is an important means to solve problems, such as insufficient medical resources, rising treatment costs, and poor medical conditions, which are caused by the growing population in the world (Hassan et al., 2017; Abidi et al., 2020; Liu et al., 2021). As an emerging technology, BAN has aroused an immense interest in academia and industry since its inception. In 2012, the Institute of Electrical and Electronics Engineers (IEEE) published an official standard, IEEE 802.15.6: Wireless Body Area Networks (Standard, 2012). As shown in Figure 1, the said standard defines a human-centered wireless communication network as consisting of sensor nodes and related devices placed on or inside the human body (Pandey et al., 2019). Due to the particularity of BAN, the network transmits users' privacy information, and consequently, any unauthorized access or illegal data tampering will cause major problems to users; therefore, BAN has set high requirements for security (Shen et al., 2018; Hajar et al., 2021). However, the BAN system is limited in resources and is sensitive to the energy consumption of sensor nodes; therefore, most of the existing network security schemes are not applicable (Shi et al., 2012; Qadri et al., 2020). Therefore, designing a security solution with low power consumption and high-intensity security that meets the requirements of a BAN system to realize that data security has become a major challenge in the field of BAN research (Narwal and Mohapatra, 2021).

Researchers have proposed a variety of ways to improve the safety of BAN data. The first way for data encryption is the conventional symmetric encryption scheme. The advanced encryption standard (AES)-based encryption method for BAN data has been proposed in the study of Gangadari and Rafi Ahamed (2016). The second-order reversible one-dimensional cellular automata are used to replace the lookup tables in the AES algorithm to generate an S-box for the nonlinear substitution of data. The experimental results have shown that this scheme includes better security features than the conventional AES algorithm. However, generating the encryption key requires adequate amounts computation, and the BAN system uses more energy due to data encryption. Therefore, the conventional symmetric encryption scheme is not suitable for a BAN system (Bai et al., 2019; Wang et al., 2020). The second way for data encryption is based on the physiological sign information in a BAN system. Different from common sensor nodes, the sensor nodes in BAN collect human physiological sign parameters. These parameters will present different characteristics according to different individuals. Even for the same individual, the information on physiological signs is time-varying. Therefore, these parameters collected from the human body can be used for data security in a BAN system (Al-Janabi et al., 2017). Compared with traditional data encryption schemes, these key generation methods can effectively reduce energy consumption in the process of encryption (Liu et al., 2013; Mainanwal et al., 2015). In their study Wang et al. (2011) proposed a method to extract characteristic values of electrocardiogram (ECG) signals based on the implicit Markov model, and the encryption key for BAN data was generated by combining the hash function. This scheme does not require key distribution and strict time synchronization. Fast Fourier Transform (FFT) is performed on an ECG signal and photoplethysmogram (PPG) signal, respectively, as described in the study by Venkatasubramanian et al. (2008) and by Ramli et al. (2013), and the generated characteristic values and fuzzy algorithm are used to construct a security mechanism for BAN. In his study, Moosavi (2021) puts forward two different key generation methods based on the PPG signal to improve the security of BAN. The first method is realized using the Galois linear feedback shift register (LFSR) and a continuous interbeat interval (IBI) of the PPG signal. The second method is realized using the IBI as the seed generator for the AES algorithm. Compared with the existing methods that only rely on the IBI sequence, this method provides better random performance. In the study by Faragó et al. (2019), a time-domain technique based on the cross-correlation has been proposed to evaluate the characteristics of ECG, PPG, or electromyography (EMG) signals for the identification and authentication in BAN systems. This method can complete the security authentication of nodes without increasing the calculation amount of BAN nodes. In this article, we propose a security scheme based on the recognition of EEG characteristics and the LFSR method for BAN. The random EEG signal characteristics containing individual differences were calculated as the input data for MD5 to solve the security problem of the key. The simple structure of the LFSR was used to generate a random stream key, which improves the data encryption strength of a BAN system.

The remainder of this article is organized as follows: Section 2 puts forward the EEG signal and the characteristic value extraction method. Section 3 presents the initial key generation method based on MD5. Section 4 provides the generation method of keys based on the LFSR. Section 5 presents the experimental results of the encryption data and provides the analysis. Finally, the conclusion of the security scheme is given in Section 6.

## 2. EEG signal acquisition and characteristic value extraction method

### 2.1. EEG signal acquisition

Electroencephalogram (EEG) is a method for recording the effect of electrical activity on the nerve cells in the brain to reflect the brain activity in a human being. The human brain is made up of tens of thousands of neurons, and the EEG signals are the electrical signals that are generated by the activity between these neurons (Lotte et al., 2018). The single-lead EEG has poor determinacy and strong randomness, thus the nonlinear research is limited to some extent and the recognition results are poor. While the multi-lead EEG contains more information concerning the human brain, it can better re?ect the overall information on brain activity (Galderisi et al., 1996).

It is difficult to accurately characterize the electrical activity of the human brain because the EEG is characterized by a strong background noise, a weak signal amplitude, a strong non-stationarity, and randomness. The concept of frequency was first used to describe the electrical activity in the brain in 1929. The frequency of electrical activity in the human brain varies between 0.5 Hz and 30 Hz. At present, it is generally accepted that the range of EEG frequency variations is divided into four frequency bands, which are: (1) δ wave: frequency is 0.5–4 Hz and the amplitude is 20–200 μV. (2) θ wave: frequency is 4–7 Hz and the amplitude is 20–150 μV. (3) α wave: frequency is 8–13 Hz and the amplitude is 20~100 μV. (4) β wave: frequency is 13–30 Hz and the amplitude is 5–20 μV.

**Figure 2:**
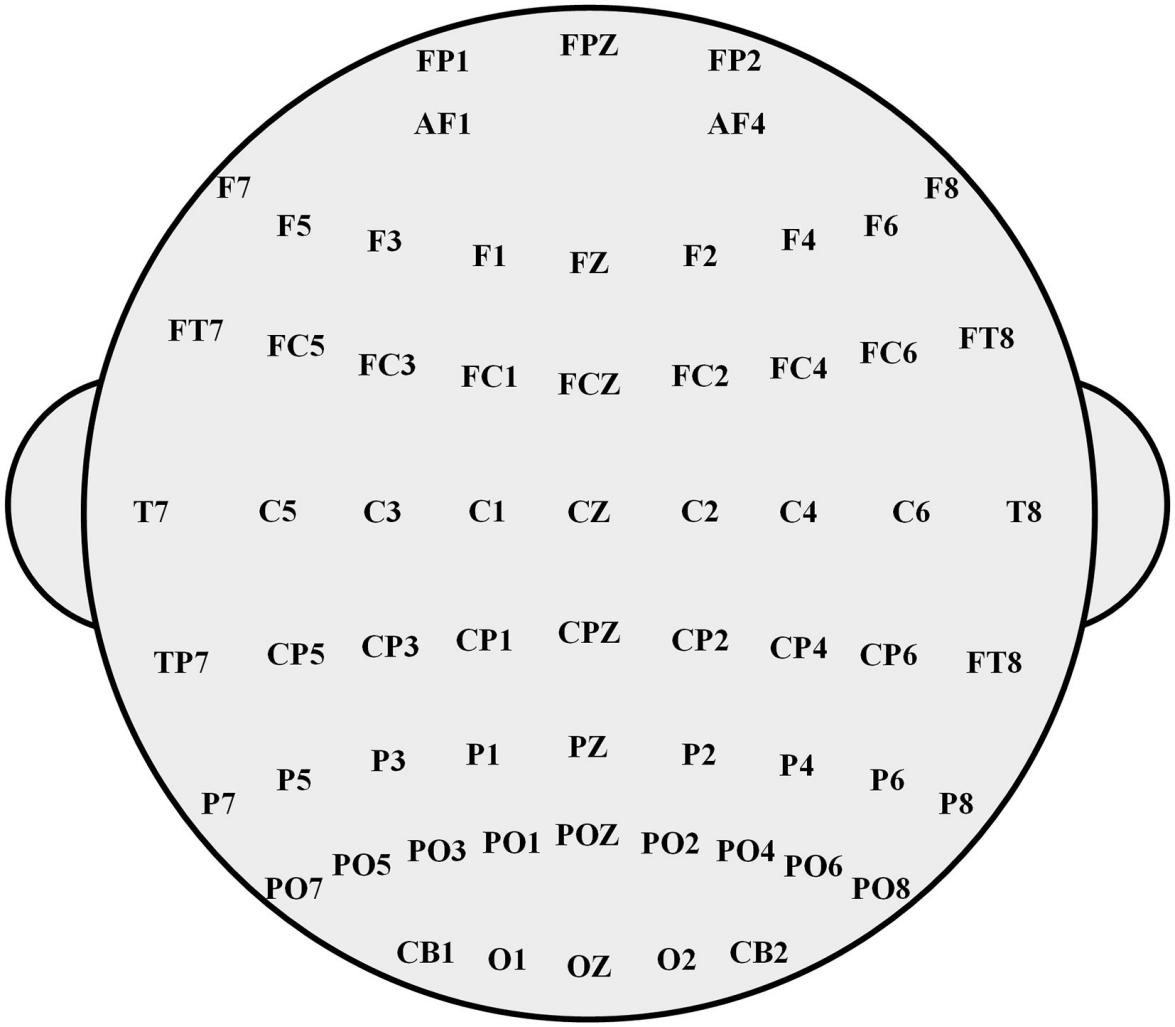
A 62-channel ESI NeuroScan system with the sampling frequency of 200 Hz.

In this study, the EEG experimental dataset SEED-IV was used for experimental testing. In SEED-IV, 15 volunteers between 20 and 24 years of age were involved in the experiment, and 72 movie clips were selected as a library of four emotions (happiness, sadness, fear, and neutral). Each volunteer was required to undergo three sessions with an interval of one week or more, and each session contained 24 trials. Each trial usually lasted ~3 min and is divided into three parts: 5 s hint of start, approximately 2 min movie clip, and 45 s emotion assessment. In the dataset, EEG signals were extracted from each volunteer at different times during the same scene and the same stimulus. As shown in Figure 2, the multi-channel original EEG signals were extracted by the 62-channel ESI NeuroScan system with the sampling frequency of 200 Hz. The other irrelevant bands were filtered with filters ranging from 0.5 Hz to 75 Hz to remove noise. Then, the time-domain and frequency-domain characteristics of EEG signals were obtained, and the EEG signals were expressed effectively (Zhang et al., 2013; Meng et al., 2014; Yang et al., 2014).

### 2.2. Extraction method of EEG characteristics

Electroencephalogram is a time-varying and non-stationary signal. Since the EEG signal was discovered, the usual method of analysis is to calculate the power spectrum of the EEG signal by Fourier transform; however, the Fourier transform is based on the premise of a stationary random process. However, the EEG signals detected in practical applications cannot satisfy the hypothesis of stationarity, such as the EEG signals in sleep, seizure, or other states; therefore only by combining time and frequency processing can better results be obtained.

The wavelet transform is equivalent to a digital microscope, including the functions of magnification, narrowing, and panning. The function of a wavelet transform is similar to that of a set of band-pass filters with an equal bandwidth and a variable center frequency. Compared with the traditional Fourier transform, the wavelet transform is a local transform of space/time and frequency, which can effectively extract information from signals, and carry out a multi-scale detailed analysis of functions or signals through operational functions such as scaling and shift, with good time-frequency characteristics. The wavelet transform can provide a frequency-varying “time-frequency” window, which automatically narrows when high-frequency information is detected, allowing for a fine analysis of the signal at higher frequencies. When a low-frequency signal is detected, the “time-frequency” window is automatically widened to allow for conducting a profile analysis of the signal at a lower frequency. The time-frequency characteristics of the wavelet transform are very suitable for analyzing the transient characteristics and time-varying characteristics of non-stationary signals.

The wavelet packet decomposition, also known as optimal subband tree structuring, is the further optimization of the wavelet transform. The concept behind wavelet packet decomposition is to use the analysis tree to represent the wavelet packet, that is, to analyze the details of the input signal using the wavelet transform with several iterations. The idea behind the main algorithm of wavelet packet decomposition is that, on the basis of the wavelet transform, in each level of signal decomposition, in addition to the further decomposition of the low-frequency subband, further decomposition of the the high-frequency subband also takes place. Finally, by minimizing the cost function, the optimal signal decomposition path is calculated, and the original signal is decomposed according to the decomposition path. Compared with the traditional wavelet analysis, the wavelet packet analysis can adaptively select the best basis function according to the characteristics of the analyzed signal after the multi-level division of the frequency band so as to make it match the signal and improve the analysis ability of the signal (Yin et al., 2008). In the wavelet packet analysis, *φ*(*t*) represents the scale function and *ψ*(*t*) represents the wavelet function, therefore:


(1) $$\begin{array}{l} \psi_{0}\left(t\right)=\varphi \left(t\right) \end{array}$$

(2) $$\begin{array}{l} \psi_{1}\left(t\right)=\psi \left(t\right) \end{array}$$

(3) $$\begin{array}{l} \psi_{2m}\left(t\right)=\overset{+\infty }{\underset{k=-\infty }{\sum }}h_{k}\psi_{m}\left(2t-k\right) \end{array}$$

(4) $$\begin{array}{l} \psi_{2m+1}\left(t\right)=\overset{+\infty }{\underset{k=-\infty }{\sum }}g_{k}\psi_{m}\left(2t-k\right), \end{array}$$

where *h*_*k*_ is the low-pass filter and *g*_*k*_ is the high-pass filter. Function {*ψ*_*n*_} is the wavelet packet of the scale function *φ*(*t*).

In this article, the wavelet packet transform theory was used to process EEG signals, and the characteristics of different EEG frequency bands (α wave, β wave, δ wave, and θ wave) were calculated. Through a multi-resolution decomposition of the wavelet packet, the optimal wavelet packet tree was selected to reconstruct the specified EEG frequency bands. In this article, a discrete Meyer wavelet was used to decompose EEG signals. The Fourier transform of the Meyer wavelet scale function is:


(5) $$\varphi \left(t\right)=\left\{ \begin{array}{l} {\left(2\pi \right)}^{\frac{1}{2}}\;\left|t\right|<\frac{2\pi }{3}\\ {\left(2\pi \right)}^{-\frac{1}{2}}\;\cos\left[\frac{\pi }{2}\nu \left(\frac{3}{2\pi }\left|t\right|-1\right)\right]\;\frac{2\pi }{3}\leq \left|t\right|\leq \frac{4\pi }{3}\\ 0\text{ else} \end{array}\right.$$

(6) $$\nu \left(t\right)=\left\{ \begin{array}{l} 0\;t\leq 0\\ 1\;t\geq 1 \end{array}\right.$$

(7) $$\begin{array}{l} \nu \left(t\right)+\nu \left(1-t\right)=1 \end{array}$$

The EEG signal was decomposed into eight layers, and the minimum frequency resolution can be estimated by formula (8).


(8) $$\begin{array}{l} f_{\min}=\frac{f_{s}}{2\cdot 2^{8}}=0.3906\text{Hz}, \end{array}$$

where *f*_*s*_ is the sampling frequency of the EEG signal and the value of *f*_*s*_ is 200 Hz.

The more the layers of wavelet packet decomposition, the higher the frequency resolution. The wavelet packet decomposition satisfies four kinds of EEG frequency band filtering requirements.

## 3. Initial key generation method

**Figure 3:**
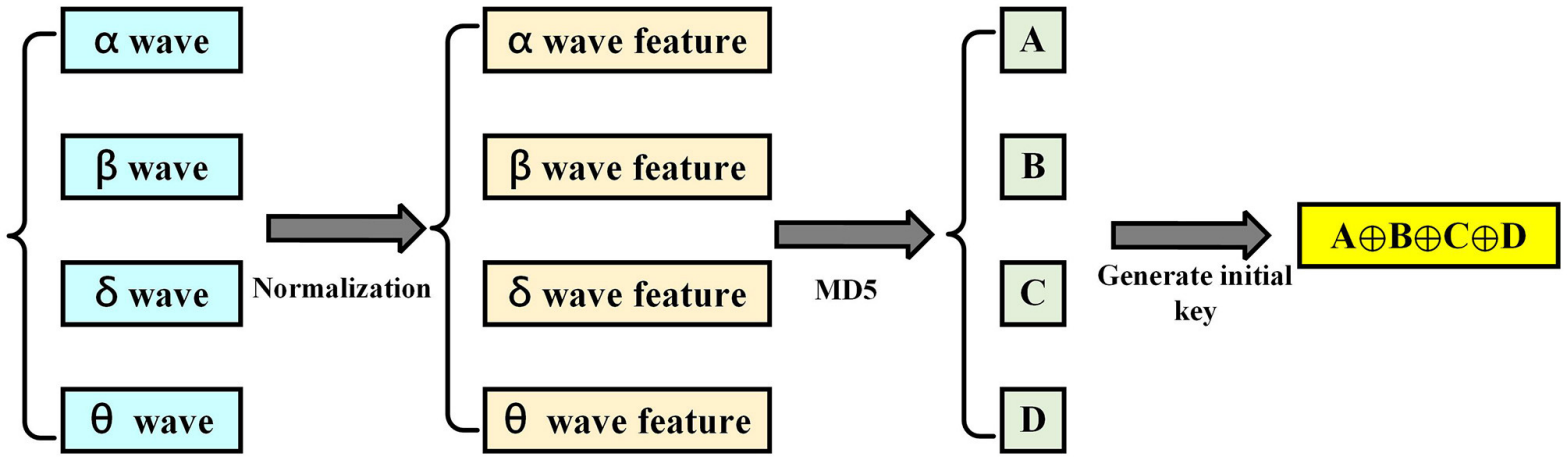
Flowchart of the key for BAN data security.

After performing the extraction method of EEG characteristics, MD5 and LFSR were used to generate keys for data encryption, as shown in Figure 3. The message-digest (MD) algorithm is a widely used password hash function, which has the following characteristics: first, the length of the output sequence calculated by the MD algorithm is fixed, regardless of the length of the input message. Second, the message digest appears randomly. In fact, different input data in the MD algorithm will result in different output sequences after calculation. Third, as the MD algorithm is a one-way function, it can only calculate the summary sequence from the input data bit can neither recover any input data from the output sequence nor find any information related to the input data from the output sequence. The MD5 algorithm generates a 128-bit (16-byte) hash sequence based on the input data. It can be used to verify the integrity and consistency of data transmission. The MD5 algorithm was announced in 1992 to replace the MD4 algorithm. MD5 treats the entire file as one large text message and generates a unique MD5 message digest through an irreversible string conversion algorithm. If anyone makes any changes to a file, its MD5 value will change.

**Figure 4:**
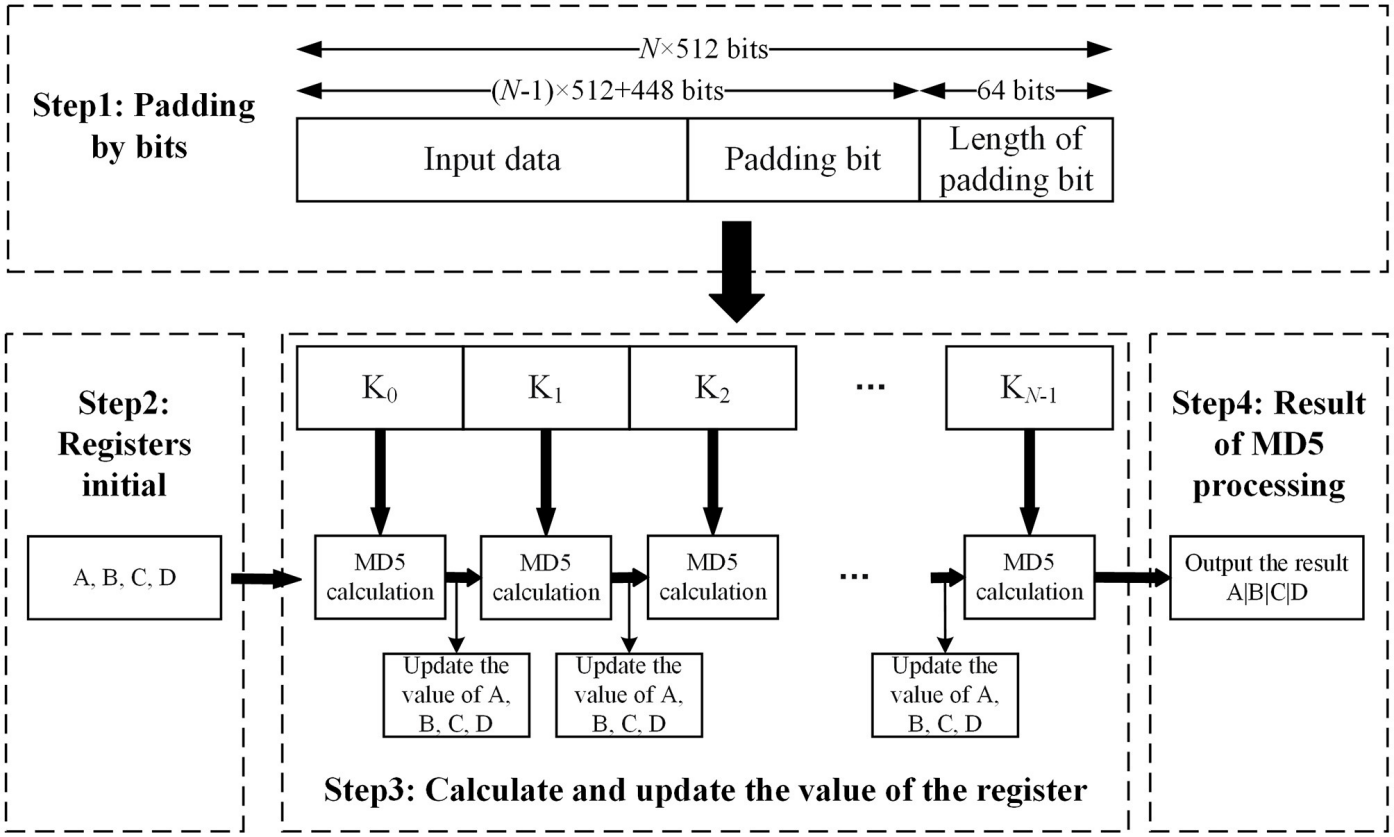
Flowchart of MD5 processing.

The principle of the MD5 algorithm is that the input data was grouped into 512 bits. Each group was then divided into sixteen 32-bit subgroups, and the grouped data were processed through a series of calculations. Finally, the 128-bit hash output sequence is composed of four groups of 32-bit sequences in sequence. Figure 4 shows the process of the MD5 algorithm. Each operation was performed by the 128-bit result value of the previous round and the current 512-bit value. The MD5 process is as follows:

(1) In the MD5 algorithm, the input data need to be padded by bits first, and the bit number of the data modulo 512 is required to be 448. Even if the bit number of the input data modulo 512 yields exactly 448, it must be padded by bits. The first bit of the padding bits is 1 and the rest is 0.
(2) The MD5 algorithm uses four 32-bit registers, A, B, C, and D, to store intermediate variables and the final result. Registers A, B, C, and D are assigned initial values at the beginning of the MD5 calculation.
(3) MD5 algorithm defines four nonlinear functions, F, G, H, and I, to process the input data after grouping. Four different functions, F, G, H, and I, are used to process each 512-bit dataset. Each round takes the data in the updated register, A, B, C, and D, and the current 512-bit dataset as input and gets the new register values of A, B, C, and D after calculation.
(4) The final values of A, B, C, and D are spliced together in the order from low-bit data to high-bit data to form the output data.

## 4. Stream key generation based in LFSR

**Figure 5:**
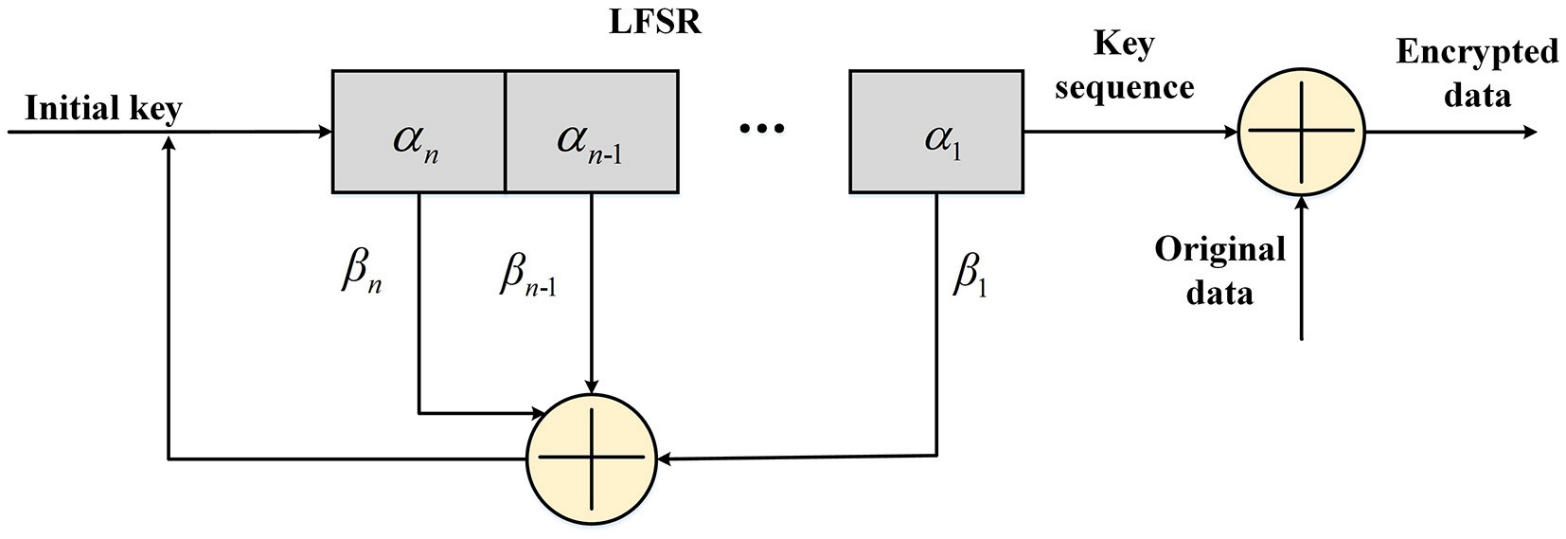
Linear feedback shift register (LFSR)-based encryption process.

Taking some sequences existing in the shift register as the input of the linear feedback function, after making certain calculations in the function, the output results were filled to the leftmost end of the shift register so that such a shift register would have a continuous output. This shift register is called the linear feedback shift register (LFSR). In fact, the feedback function in the LFSR only performs an XOR on certain bits in the shift register and populates the result to the leftmost end of the shift register, as shown in Figure 5. The data of each bit in the LFSR may or may not participate in the XOR, and the bits that participate in the XOR are called taps. An *n*-order LFSR can only traverse 2^n^-1 states at most, that is to say, the maximum period of an LFSR is 2^n^-1. The sequence generated by an LFSR with a period of 2^n^-1 is called the *m* sequence, and the *m* sequence has a higher encryption strength among the LFSR of the same level. The cipher text is calculated by the pseudorandom sequence XOR the plaintext.

The expression of the 128-order *m* sequence used for key generation is shown in formula (9).


(9) $$\begin{array}{l} f\left(N\right)=N^{127}+N^{126}+1 \end{array}$$

## 5. Experimental results and analysis

**Figure 6:**
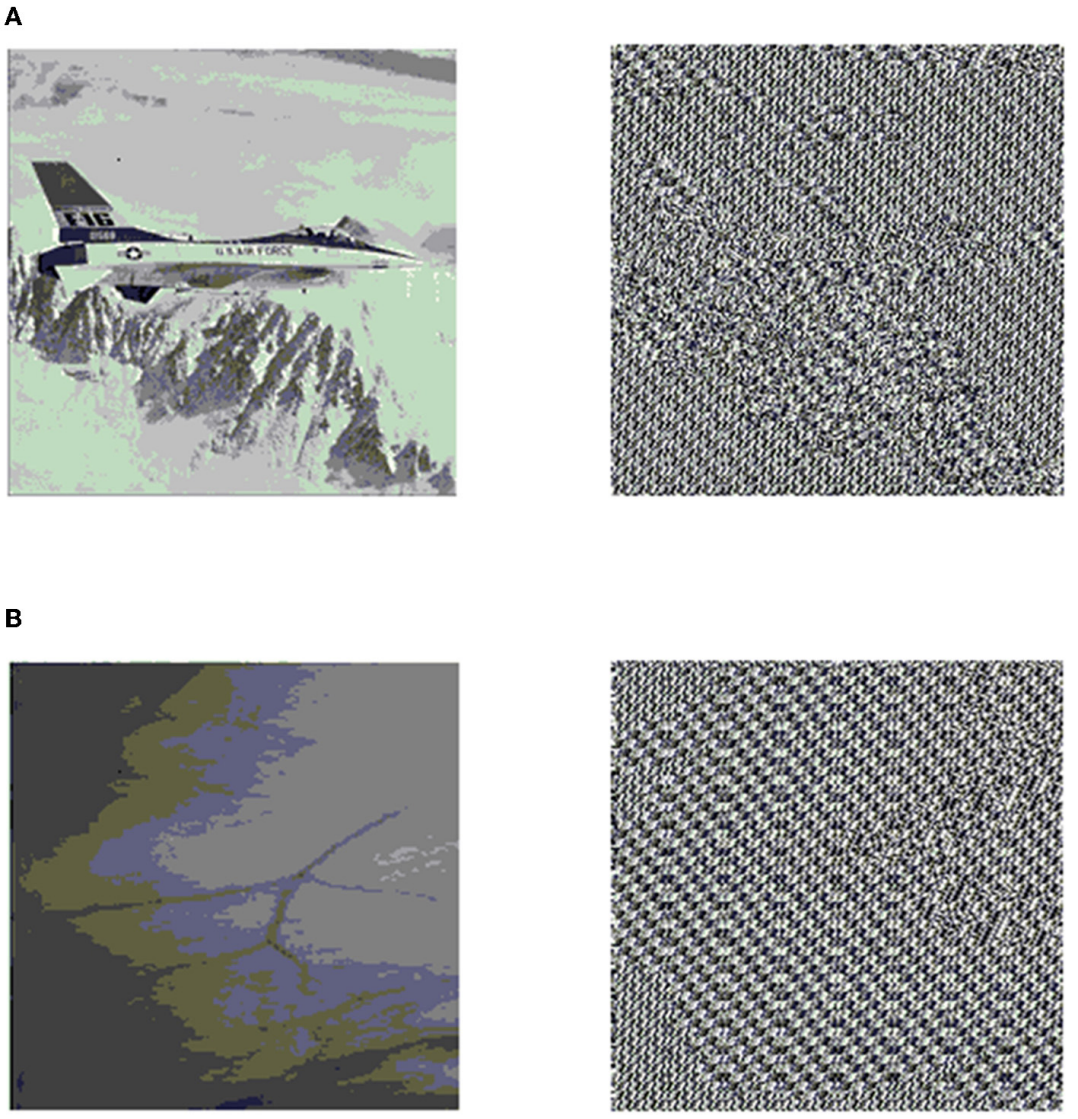
Original and encrypted images. **(A)** Airplane. **(B)** Medical endoscopic image.

To verify the designed data security scheme based on EEG characteristics and LFSR, MATLAB software is used to simulate the encryption of image data. Using image data for verification of the designed data security scheme is important because the BAN system contains a large number of images such as electromyography images, endoscopic images, and medical images. Furthermore, image data are more intuitive for verification. In this article, the image Airplane and a medical endoscopic image were used to verify the encryption effect of the proposed data security scheme. Figure 6 shows the original images and the encrypted images.

**Figure 7:**
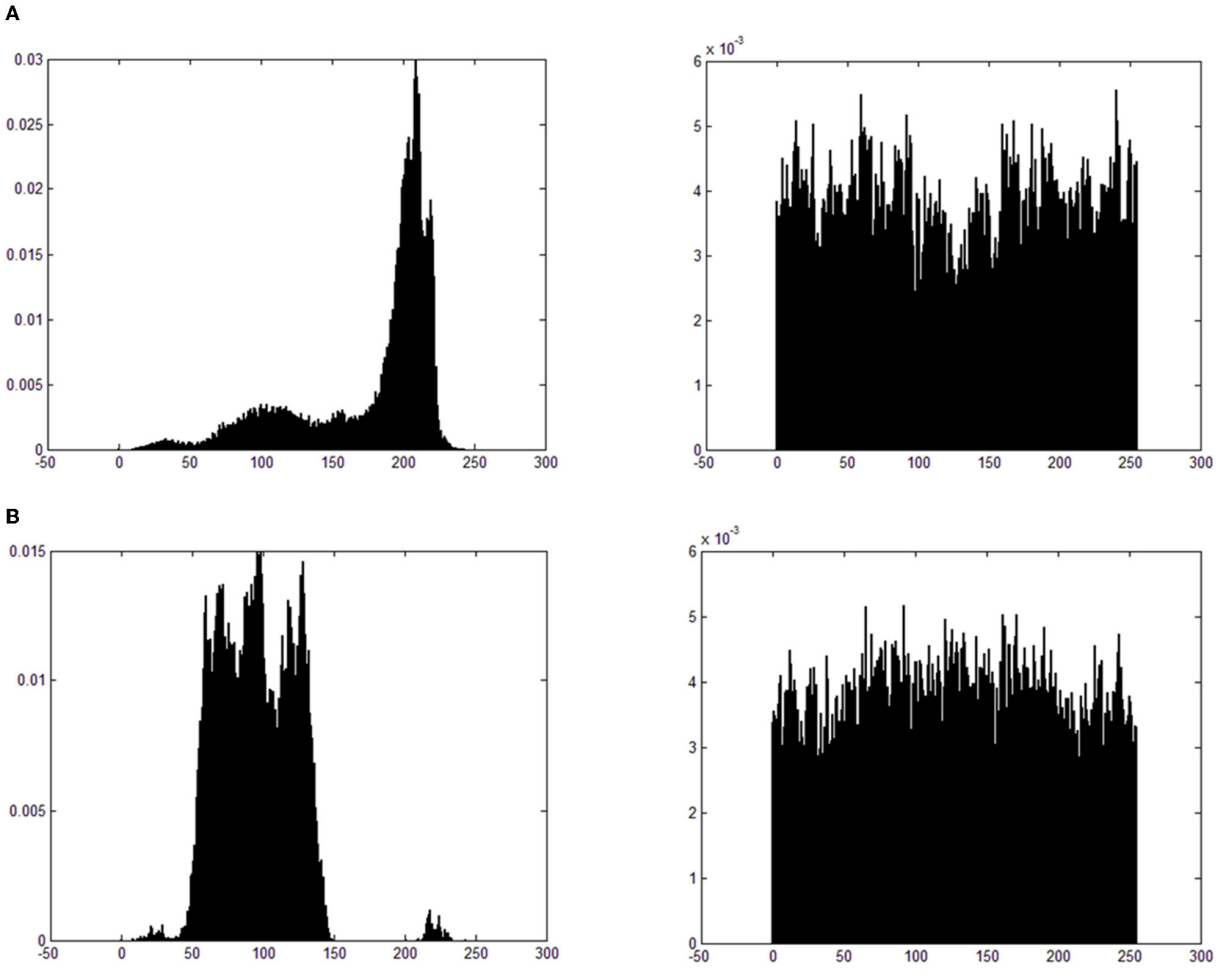
Gray histogram of the original and encrypted images. **(A)** Airplane. **(B)** Medical endoscopic image.

Gray distribution refers to the distribution of gray value of gray images, which reflects the most basic statistical characteristics of an image and is generally represented by a gray histogram. The gray value distribution of the original image is usually uneven; therefore, there is a certain probability to crack the image data by calculating the statistical features. Figure 7 presents the gray histogram results of the image Airplane and the medical endoscopic image. As shown in Figure 7A, the left gray histogram is the gray value distribution of the original image, and it is relatively concentrated. However, after the encryption is completed, the result of the distribution of the images becomes more uniform, as shown in the right gray histogram of Figure 7A. Similar to Figures 7A, B yields the same result after encryption. Therefore, through the security scheme proposed in this article, the gray value distribution of the images is scrambled and became more uniform.

The two-dimensional correlation of images is used to verify the correlation degree of the original image data and the encrypted one, and *K* is the correlation coefficient. The calculation method of *K* is given as follows:


(10) $$\begin{array}{l} \;K=\overset{M}{\underset{a=1}{\sum }}\overset{N}{\underset{b=1}{\sum }}\left(O_{ab}-\bar{O}\right)\left(E_{ab}-\bar{E}\right)/\\ \sqrt{\left(\overset{M}{\underset{a=1}{\sum }}\overset{N}{\underset{b=1}{\sum }}{\left(O_{ab}-\bar{O}\right)}^{2}\right)\left(\overset{M}{\underset{a=1}{\sum }}\overset{N}{\underset{b=1}{\sum }}{\left(E_{ab}-\bar{E}\right)}^{2}\right)} \end{array}$$

where *O*_*ab*_ and *E*_*ab*_, respectively, represent the gray values of the image Airplane and the medical endoscopic image at the point (*a, b*) before and after the encryption.

After the designed method in this article was used to encrypt the image Airplane and the medical endoscopic image, the two-dimensional correlation coefficients *K*_1_ =1.38×10^−8^ and *K*_1_ =3.24×10^−9^ of the two images before and after encryption were calculated by formula (9). It can be observed that the two-dimensional correlation coefficient approaches 0, indicating that the correlation of images before and after encryption is very low. This method can effectively encrypt image data.

To verify the correlation of adjacent pixels in the images, we select the adjacent pixel pairs in the original image and the encrypted one, and *R* is the correlation coefficient. The calculation method of *R* is shown in formula (14). Furthermore, the correlation coefficients in the vertical, horizontal, and diagonal directions of the images are calculated, respectively,


(11) $$\begin{array}{l} E\left(x\right)=\overset{N}{\underset{i=1}{\sum }}x_{i}/N \end{array}$$

(12) $$\begin{array}{l} D\left(x\right)=\overset{N}{\underset{i=1}{\sum }}\left[x_{i}-E\left(x\right)\right]/N \end{array}$$

(13) $$\begin{array}{l} \mathrm{cov}\left(x,y\right)=\overset{N}{\underset{i=1}{\sum }}\left[x_{i}-E\left(x\right)\right]\left[y_{i}-E\left(y\right)\right]/N \end{array}$$

(14) $$\begin{array}{l} R=\frac{\mathrm{cov}\left(x,y\right)}{\sqrt{D\left(x\right)D\left(y\right)}} \end{array}$$

where *x*_*i*_ and *y*_*i*_ are the gray values of the *i*th adjacent pixel pairs, *E*(*x*) and *E*(*y*) are the averages of the image data, *D*(*x*) and *D*(*y*) are the variances, which measure the deviation between the image data and its average and *N* is the adjacent pair number of the image.

The calculated results of the correlation coefficient between the original image and the encrypted image are shown in Table 1. It is worth noting that, after data encryption of the image Airplane and the medical endoscopic image, the correlation of adjacent pixels in the three directions is effectively reduced, and it is difficult for the attacker to obtain the statistical features of the plaintext, thus improving the security of the system.

**Table 1:** The correlation coefficient of the images in three directions.

**Image name**	***R***	**Vertical direction**	**Horizontal direction**	**Diagonal direction**
Airplane	Original	0.8834	0.9251	0.9118
	Encrypted	0.6247	0.2763	0.5570
Medical endoscopic image	Original	0.9251	0.9472	0.9346
	Encrypted	0.5896	0.3107	0.5713

## 6. Conclusion

To meet the requirements of low power consumption and high-intensity security in a BAN system, this article proposes a data security scheme that is based on EEG characteristic values and LFSR construction. To be specific, the characteristics of EEG signals are mined by the wavelet packet transform, and α, β, δ, and θ waves are constructed to effectively characterize the features of EEG signals, which are input into the MD5 system through normalization, and the initial key is calculated. Then, a 128-order *m* sequence is used to generate a stream key to encrypt privacy data in BAN. Finally, a variety of evaluation results prove that the proposed security scheme has enough ability to ensure data security in BAN. The BAN security scheme in this article only considers the method of data encryption; however, it does not consider the scheme of node authentication, which has certain limitations. In the future study, this project will invest more research in the following aspects. According to the relatively simple hardware structure of LFSR (m sequence), the advantages of the proposed method are further verified. Based on the characteristics of the BAN system, other types of feature extraction and calculation methods, such as ECG, PPG, and gait, will be studied to determine whether they are suitable for data security. There are still some important security problems in BAN that need to be solved. Therefore, the solutions for authentication with low power consumption and high reliability in BAN systems will be investigated by us.

## Data availability statement

Publicly available datasets were analyzed in this study. This data can be found at: https://bcmi.sjtu.edu.cn/home/seed/seed-iv.html.

## Author contributions

TB: conceptualization, methodology, writing—original draft, and writing—review and editing. JL: design of the methodology and validation of the experiments. YJ: validation and editing. JY: supervision. YD: validation. All authors contributed to the article and approved the submitted version.
